# Multiple drill-hole osteotomy in hand surgery – description of a novel application and proof of feasibility

**DOI:** 10.1186/s12891-017-1895-4

**Published:** 2017-12-13

**Authors:** T. Haider, D. Geisler, G. Thalhammer, J. Erhart

**Affiliations:** Department of Trauma Surgery, Medical University of Vienna, Waehringer Guertel 18-20, A-1090 Vienna, Austria

**Keywords:** Osteotomy, Corrective osteotomy, malunion of phalangeals, Malunion of metacarpals, Multiple drill-hole osteotomy

## Abstract

**Background:**

Malunion of phalangeal and metacarpal bones are often associated with impairment of hand function and pose a challenging task for treating surgeons in most cases. When applicable, corrective osteotomy is the treatment of choice, where the affected bone is cut to correct malalignment using chisels or saws. The use of these instruments is associated with several drawbacks especially in hand surgery. We aimed to determine whether a multiple drill-hole (MDH) osteotomy technique was suitable for performing corrective osteotomies of metacarpal and phalangeal bones.

**Methods:**

This case series included 11 patients with malalignments or malunions of phalangeal or metacarpal bones. Corrective osteotomy was performed with the MDH technique. Follow-up examinations included clinical evaluations and radiography at frequent intervals, between 2 and 22 months postoperatively.

**Results:**

In all cases, planned osteotomies were technically feasible with the MDH technique. Apart from one case of a broken drillbit, no intraoperative or postoperative complication was recorded. All performed osteotomies healed within a mean of 6 weeks to radiological consolidation. In all cases, satisfactory results were achieved.

**Conclusion:**

The present study was the first to test MDH osteotomy for hand surgery. We demonstrated that MDH was feasible for corrective osteotomies of metacarpal and phalangeal deformities. Advantages included excellent feasibility for osteotomies performed at varying angles, precise execution, reduced risk of collateral damage, and flexibility for performing intra-articular osteotomies.

## Background

Metacarpal and phalangeal bone deformities pose dreaded complications when the bone fractures. The various potential deformities are termed rotational, flexion, extension, and impaction. These deformities result in functional deficits and aesthetic problems, in many cases [1, 2].

In reconstructive orthopedic surgery, the multiple drill-hole (MDH) osteotomy is an established technique for treating malpositions of large bones. MDH was previously described and utilized for corrective and lengthening osteotomies of the tibia, corrective osteotomies of the radius, and correction of the Madelung deformity [3, 4]. Compared to standard osteotomies performed with an oscillating saw, the advantages of MDH include reduced damage to the periosteal layer, the ability to perform the osteotomy percutaneously, higher precision with improved control, less potential for collateral damage to adjacent neurovascular structures, lower heat generation, no constraint on the course of the osteotomy and greater contact surface at the osteotomy site [4]. One study also reported improved bone consolidation after MDH osteotomy, compared to an osteotomy executed with an oscillating saw [5]. Alternatively, dentofacial surgeons utilize an ultrasound scalpel to perform osteotomies; this technique was recently adopted for hand surgery with satisfactory results. The features and potential advantages of that technique are similar to those of the MDH osteotomy, but it has the self-evident disadvantage of requiring equipment not commonly used in orthopedic surgeries [6, 7].

Corrective osteotomy in hand surgery represents a possible treatment option in patients with malpositioned metacarpals and phalangeal bones, but only data from small case series are currently available in the literature [1, 2, 8–10]. Several osteotomy techniques have been proposed in past years, including transverse, oblique, open-wedge, closing wedge, pivot, and step-cut osteotomies. These techniques commonly utilize an oscillating saw to execute the osteotomy [1, 2, 8–10]. However, oscillating saws are associated with several disadvantages, including potential damage to nerve and soft tissues, limited control of the osteotomy, and heat generation. The close proximity of anatomical structures around the metacarpals and phalanges intensifies these problems [6]. Therefore, we tested the MDH osteotomy technique for performing corrective osteotomies that targeted malpositions of metacarpal and phalangeal bones.

To our knowledge, the present study was the first to describe MDH osteotomies as a potential surgical treatment for malalignment of metacarpals and phalanges. This report aimed to provide proof of the feasibility of the MDH osteotomy for treating malpositions of phalanges or metacarpal bones in a case series of 11 patients.

## Methods

This study was approved by the local ethics committee of the Medical University of Vienna. This retrospective observational study included 11 patients that underwent MDH osteotomies of metacarpals or phalanges. The present study did not affect clinical procedures. The local ethics committee of the Medical University of Vienna waived the need to obtain informed consent. Detailed patient and osteotomy characteristics are listed in Table 1.

**Table 1:** Patient characteristics. 1 = proximal, 2 = middle, 3 = distal, P = phalanx, MC = metacarpal

Patient	Injury-Surgery-Interval (months)	Finger	Osteotomy localization	Type of ostetomy	Time of surgery (min)	Type of osteosynthesis	Time to consolidation (weeks)	Follow-up (months)
1	2	index	P2 base	closing-wedge	135	plate and screws + autograft	6	2
2	4	middle	P2 base	intra-articular	145	plate	4	4
3	4	thumb	MC	dome	145	plate	8	3
4	8	middle	P2 base	intra-articular	180	plate	4	13
5	18	little	MC	dome	85	plate	12	22
6	10	little	MC	open-wedge	85	plate	4	8
7	3	ring	P1	derotational	70	plate	4	3
8	6	index	P2	intra-articular	85	screws	4	12
9	15	index	MC	modified scarf	215	screws	8	2
10	4	middle	hand reduction	hand reduction	170	plate	8	2
11	2	index	P1 base	open-wedge	105	plate + autograft	4	2
mean	7				133,5		6	6

### Location of osteotomy

MDH corrective osteotomies were performed on the proximal phalangeal bone in two patients, on the middle phalangeal bone in four patients, and for metacarpal deformities in four patients. We also used the MDH osteotomy to perform hand reduction surgery in one case, following traumatic amputation of the middle finger.

### Types of osteotomy

With the MDH technique, we performed derotational osteotomy in two patients (patients #1 and #7); articular osteotomy in three patients (patients #2, #4, and #8); dome osteotomy in one patient (patient #3); a combination of open-wedge osteotomy and dome osteotomy in two patients (patient #5 and #6); a modified scarf osteotomy in one patient (patient #9); an open-wedge osteotomy with transplantation of an autologous tricortical iliac crest autograft in one patient (patient #11); and secondary hand reduction surgery in one patient (patient #10).

### Preoperative planning

Detailed preoperative evaluations of hand function and malformation extent were carried out prior to every planned osteotomy. X-rays in at least two planes of the affected hand were acquired for every patient. Additionally, preoperative computed tomography scans were performed in two cases (patients #4 and 9), and additional preoperative magnetic resonance imaging was performed in one patient (patient #3). Prior to surgery, each patient was informed in detail about the planned surgical procedure, possible alternative treatments, potential risk factors, and postoperative protocols. Written informed consent was obtained from all patients.

### Surgical approach

Depending on the location of the deformity, we used three different approaches. For an MDH osteotomy of a non-articular malposition of the phalanges, we selected a standard dorsal approach. Briefly, following skin incision, a longitudinal split of the extensor tendon was performed to expose the targeted part of the bone. For a metacarpal osteotomy, the respective extensor tendons were mobilized and retracted aside to expose the osteotomy site. Subsequently, the periosteal layer was cut longitudinally and sharply dissected from the bone to permit thorough closure of the periosteal layer at completion of the osteotomy; this closure allows the extensor tendon to glide properly over the osteotomy site. For an articular malposition at the base of the middle phalanx (patients #2 and 4), we used a shotgun approach to expose the osteotomy site [11].

### Multiple drill-hole osteotomy and osteosynthesis

**Fig. 1:**
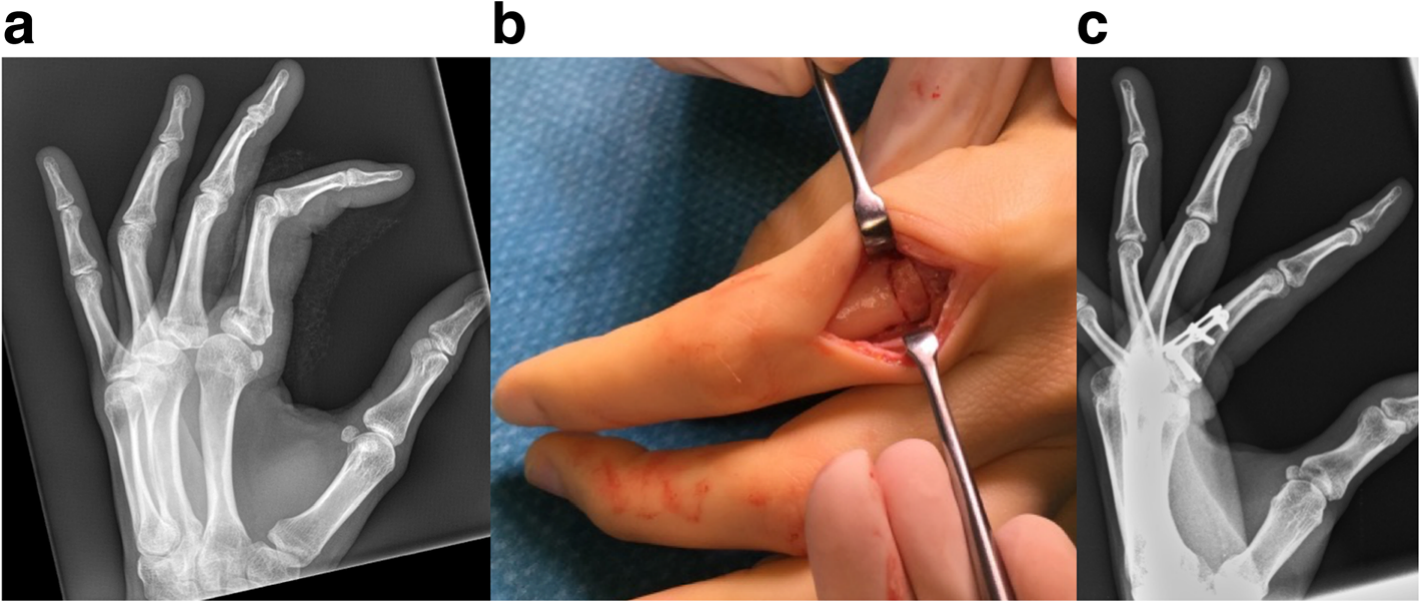
Pat. #11: **a** Pre-operative radiographic study. Patient suffered from an extension deformity following a fracture of the proximal phalanx of the index finger. **b** After multiple-drill hole osteotomy an iliac crest autograft was inserted followed by plate osteosynthesis. **c** Radiographic study 1 month post-operative

Each osteotomy was realized with surgical drills fitted with drillbits between 0.7 and 1.2 mm in diameter. In some cases, instead of a drillbit standard K-wires between 0.7 and 1.2 mm in diameter were used to perform multiple drill-holes. The osteotomy was completed by cutting the bone between the holes (connecting the holes) with a small chisel or scalpel. When feasible, we preferred completing the osteotomy with a scalpel, because it provided more control than a chisel. After correction of the malalignment under fluoroscopy guidance, an osteosynthesis was performed, depending on the case. Osteosyntheses were performed with screws, plates, or a combination of both (Table 1). When a plate osteosynthesis was planned, the plate was fixed distal to the osteotomy site, prior to execution of the osteotomy. After performing the osteotomy, the distal fragment with the mounted plated was then reduced under fluoroscopy guidance and fixed to the proximal fragment. This procedure improved control of the malalignment correction. In two cases, autologous cancellous bone was used, either from the iliac crest (patient #1) or from the olecranon (patient #2); in another case (patient #11), a tricortical iliac crest autograft was placed to correct the deformity, followed by fixation with a plate-osteosynthesis (Fig. 1). In this case, we performed the osteotomy prior to fixation of the plate, to allow introduction of the tricortical autograft.

### Osteotomy technique

**Fig. 2:**
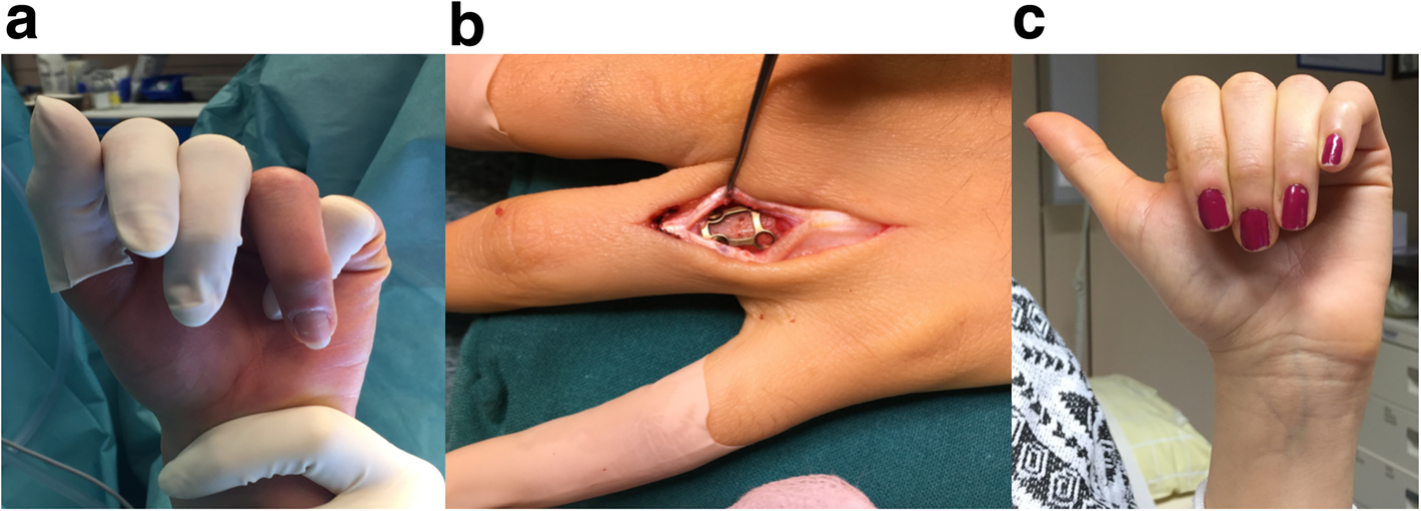
Pat. #7: **a** Preoperative clinical situation with malrotation of the ring finger; **b** Multiple drill hole osteotomy with dorsal approach. The plate was fixed on the distal fragment prior performance of the osteotomy. **c** Clinical image 1 month postoperative

Depending on the deformity, different osteotomy types were chosen. In patients #2 and #4, we used the MDH osteotomy to address an impression deformity at the base of the middle phalangeal bone. After drilling, we remodeled the articular surface with a small pestle introduced distally, inserted a cancellous bone autograft derived from the ipsilateral olecranon, and stabilized the osteotomy site with a plate-osteosynthesis. In one case (patient #2), we additionally fixed the proximal interphalangeal joint temporarily with one K-wire and removed it 6 weeks postoperatively with local anesthesia. Patient #8 had an impression deformity in the distal middle phalanx. Multiple drill holes were placed along the malunited fracture, and the fragment was then pushed distally and fixed with two screws. To correct flexion deformities in the metacarpals of three patients (patients #3, #5, and #6), a dome osteotomy was performed. In patients #5 and #6, the dome osteotomy was modified to a combination of the dome and open-wedge osteotomy to achieve the intended correction. Following completion of the dome-shaped osteotomy, the distal part was then straightened and fixed with a plate osteosynthesis, which allowed correction without shortening. A standard, open-wedge osteotomy was performed in one patient (patient #11) to correct a diaphyseal malposition of the proximal phalanx. Additionally, in this case, a tricortical iliac crest autograft was transplanted. To address a rotational deformity of phalangeal bones, we performed a derotational osteotomy in two patients (patients #1 and #7, Fig. 2). On top of the planned osteotomy site, at a dorsal site, a plate was initially fixed distal to the intended osteotomy. Then, along an oblique line, multiple drill holes were placed and connected with a scalpel to complete the osteotomy. Following correction of the rotation, the plate was then fixed with screws at a proximal site. In one patient (patient #9), we used a modified scarf osteotomy to correct a malposition of the head of the second metacarpal bone. The intended osteotomy ran dorsally from the extra-articular radial aspect of the distal metacarpal to the ulnar aspect of the metacarpal shaft. The osteotomy was approximately 2.5 cm long, and was it marked on both ends with K-wires. Then, multiple drill holes were placed and connected with a scalpel to complete the osteotomy. The distal part was then rotated dorsally to correct the malposition.

### Postoperative procedure

The postoperative protocol included early active and passive movements, under the guidance of trained hand therapists. Suture removal was performed 10 to 14 days after surgery. Postoperative clinical follow-up examinations were performed regularly, and they included documentation of range of motion and radiography.

## Results

In this case series, 11 patients (5 female, 6 male; mean age at the time of surgery: 31.5 years, range 18 to 60) received MDH osteotomies to correct malpositions of phalangeal or metacarpal bones (Table 1). The mean time between the initial injury and the correction osteotomy was 7 months (2–18 months). The mean operative time was 133.5 min (85–215). Indications for MDH osteotomy included malalignments after fractures (10 cases) and hand reduction surgery after amputation of the middle finger (1 case). The types of osteotomies were a derotational osteotomy, open-wedge osteotomy, closing-wedge osteotomy, modified scarf osteotomy, intra-articular osteotomy, and dome osteotomy. All planned osteotomies were technically feasible without complications, aside from the breakage of one drillbit (0.8 mm) during drilling (patient #9). The surgeon was able to remove the broken drillbit in total, and the osteotomy was completed as planned. In one case (patient #9), the entire extent of the planned osteotomy was not realizable, due to an underestimation of soft tissue contracture in the metacarpophalangeal joint. Nevertheless, a partial procedure provided a satisfactory result for the patient. We used both chisel and scalpel to complete osteotomies. These techniques were comparable, in terms of feasibility and outcome, with no complication. When possible, we preferred the use of a scalpel, because it provided more control in completing the osteotomy.

In this case series, all osteotomies healed with a mean time of 6 weeks, between surgery and radiologically visible bone consolidation, defined as an undetectable fracture cleft. We did not observe any surgery-related, post-operative complication. All patients reported satisfactory results at the final examination.

## Discussion

With this case series, we provided the first evidence that MDH osteotomies were feasible and safe in corrective surgery for phalangeal and metacarpal bones. Despite the small number of patients treated in this study, we are confident that our findings provide additional information and treatment options to surgeons treating cases of phalangeal and metacarpal malpositions.

In general, corrective osteotomies of phalangeal or metacarpal bones represent a rare indication in hand surgery. These procedures require a high degree of individual planning, due to the broad spectrum of malalignments and various treatment options. Taken together, these characteristics require a variety of surgical techniques, which are adopted from reconstructive orthopedic surgery [1, 2, 4, 8–10]. Previous studies showed that the MDH osteotomy technique provided several advantages over osteotomies performed with oscillating saws. In studies on reconstructive surgery of the “long bones”, the MDH technique minimized the surgical approach and reduced heat generation typically incurred with saws to optimize callus distraction. We found that, in hand surgery, MDH provided the advantages of higher precision and control during the osteotomy [6, 7, 12]. This feature was particularly important in hand surgery, which involves delicate anatomical structures in close proximity; additionally, well-orchestrated hand movements demand anatomically aligned metacarpal and phalangeal bones with small tolerance margins. Therefore, in this region, osteotomies require exact planning and execution, high precision, and strict control. In addition, the greater control of MDH increased the safety of the procedure by limiting damage to surrounding neurovascular structures. Another alternative to oscillating saws is the chisel for performing osteotomies. However, using a chisel alone for osteotomies has clear disadvantages, compared to the MDH method, including the unpredictability of the extent of osteotomy; the risk of incurring adjacent fractures; the risk of mechanical damage to tendons, ligaments, and neurovascular structures; the risk of causing bone defects at the osteotomy site; and its impracticability for performing intra-articular osteotomies. Finally, the MDH technique makes other osteotomy types possible; for instance, osteotomies that do not require bone modifications, like autologous bone transplantation or bone shortening.

Using drilling to perform osteotomies adds indentations to both planes of the osteotomy, and it increases the contact surface area, which can potentially improve initial stability. Both these features are prerequisites of enhanced bone healing [4]. We experienced subjective locking-in of both osteotomy borders due to the profile created by the multiple drill-holes, when we brought them together after correcting the malalignment. This feature provided improved control over temporary fixation prior to osteosynthesis. Sparing the periosteum requires high healing capacity at the osteotomy site. The MDH technique promotes healing by reducing the heat production and increasing the contact surface area, which is achieved with the indentions that naturally occur during drilling. Furthermore, compared to oscillating saws, the drill permits osteotomies with variable courses and angles; for example, the dome osteotomy performed in patient #3. In this case, the alternatives included a standard closing-wedge osteotomy, which would have significantly shortened the thumb, or an open-wedge osteotomy, which required the transfer of an iliac crest autograft in the diaphyseal bone. Also, compared to oscillating saws, drills cause less severe bone defects, which are inherent in the execution of all osteotomies. The reduced bone loss incurred with the MDH technique facilitated the execution of intra-articular osteotomies, as performed in patients #2 and #4. In these cases, the alternative would have been a hemi-hamate arthroplasty, which is associated with morbidity at the extraction site [13].

We suggest that there are two reasons why MDH osteotomies have not prevailed in patients suffering from metacarpal or phalangeal malunion has three reasons: 1) Orthopedic surgeons are accustomed to the use of saws for osteotomies in general, 2) Correction of metacarpal or phalangeal malunions is a rare indication, which results in reduced willingness to apply different techniques, 3) the use of a saw for osteotomy is easier in terms of technical feasibility and quicker. Even more so, the present study adds important knowledge to the field of corrective osteotomy for phalangeal and metacarpal malunion.

**Fig. 3:**
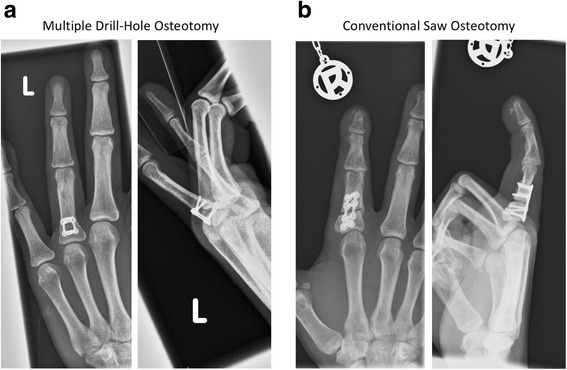
Comparison of Multiple Drill-Hole osteotomy (**a**) and conventional saw osteotomy (**b**). Pat. #7 treated with MDH osteotomy, at follow-up 3 months post-operative with good bony consolidation (**a**) compared to a patient treated with saw-osteotomy, radiographic study 3 months post-operative (**b**)

In general, a non-union represents a potential complication in osteotomies [14, 15]. The current literature demonstrates that the technique used in ulnar-shortening osteotomies was correlated with the complication rate. One study showed that oblique osteotomies were associated with lower non-union rates. Those authors claimed that increasing the contact surface at the osteotomy site resulted in improved healing rates [14, 16–18]. Heat generation during osteotomy is thought to be associated with reduced healing capacity [14, 19]. Inherently, MDH osteotomies both reduce the heat generation and increase the contact area. In our opinion, these features were involved in the satisfactory results and appropriate bone healing achieved in the present case series (Fig. 3). Only in one case (patient #5), we observed a prolonged period of bone consolidation of 12 weeks. In this case, a combination of dome- and open-wedge osteotomy was necessary to correct the underlying malposition. To avoid shortening, a remaining bone-gap after osteosynthesis was required leading to longer time to consolidate in this case. Still, to our opinion bone healing was achieved in an adequate timeframe comparable to other techniques. Recent studies have shown that, aside from the rotational speed and the feeding rate (i.e. forward momentum of the drill within the bone), the drillbit diameter is a key factor associated with heat production during drilling [20, 21]. In one study, critical heat production was reported with drillbits that were 4.5 mm in diameter, and drilling with smaller diameter drillbits did not lead to critical heat production [21]. In the present study, we used drillbits up to 1.2 mm in diameter. Augustin et al. reported a maximum temperature of 41.5 °C when drilling with a 2.5-mm drillbit, which is well below the critical temperature (47 °C) for thermal osteonecrosis [21, 22]. Those authors also showed that the drill angle was not relevant for heat production during drilling. Those findings supported our findings, and underlined the advantages of using MDH osteotomy in hand surgery. Also, we only experienced surgery-associated complication in one case of drillbit breakage (patient #9) using a drillbit of 0.8 mm in diameter. In this case, we were able to remove the broken drillbit easily. To avoid this complication, cautious drilling is important especially when using small diameter drillbits. The osteotomy site needs to be dissected meticulously to avoid drillbit deflection by soft tissue, which is associated with increased risk of drillbit breakage.

Short time intervals between surgery and bone consolidation make it possible to implement early active postoperative therapy protocols, which are associated with earlier returns to work. Based on our experience with osteotomies, achieving radiologically visible bone consolidation within a mean of 6 weeks is a comparatively short healing time for osteotomies, in general. This healing time was comparable to that found in current studies on MDH and to that observed in naturally occurring bone consolidiation [23, 24].

Recently, patient-specific instruments and drill guides were described for treating complex malunions of the distal radius with MDH ostetomy [24, 25]. Further development of this and similar techniques, including patient-specific drill sleeves, screws, and plates, might be applicable to hand surgery in the near future; moreover, these developments might lead to individualized osteotomies.

This small, retrospective case series had some limitations. We could not provide proof that MDH osteotomies were superior to osteotomies performed with oscillating saws, due to the study design. Future prospective clinical trials would be required to address this question. However, given the small patient numbers, in general, and the number of cases that require specific individual treatment strategies, it is unlikely that such prospective clinical trials will be feasible. Fortunately, the present case series includes important information for surgeons that treat these rare cases of phalangeal and metacarpal malalignment.

## Conclusion

The present observational case series study demonstrated the feasibility and safety of using MDH for correction osteotomies in hand surgery. Our results suggested that MDH osteotomy represented an attractive alternative to standard procedures with oscillating saws or a chisel alone for correction osteotomies of phalangeal and metacarpal bones. In particular, for complex cases, in our opinion, the MDH method can extend the spectrum of potential surgical procedures.

## Abbreviations

MDH: Multiple-drill hole
